# Supplementary material for: Febrile Children with Pneumonia Have Higher Nasopharyngeal Bacterial Load Than Other Children with Fever
Source: Pathogens. 2023 Mar 27;12(4):517. doi: 10.3390/pathogens12040517 (PMC10143154; doi:10.3390/pathogens12040517)
Supplement: Supplementary file 1 [file pathogens-12-00517-s001.zip › pathogens-2217110 SM.pdf]

Table S1. Target genes and primer and probe sequences used for bacterial qPCR assays. BHQ: Black Hole Quencher.

Organism	Gene	Forward Primer Sequence 5′ - 3′	Reverse Primer Sequence 5′ - 3′	[Dye]ProbeSequence 5′ - 3′
S. pneumoniae[33]	lytA	ACGCAATCTAGCAGATGAAGCA	TCGTGCGTTTTAATTCCAGCT	[Cy5]GCCGAAAACGCTTGATACAGGGAG[BHQ2]
M. catarrhalis[34]	ompJ	CAGCCTAGCAGGCGGTGTT	TTGCTTCAACGCCCACATT	[VIC]TTGGCTTTAAACCATTAGC[MGB]
H. influenzae[11]	hdp	TTGGCCCAGGTTGGTATATG	TTACGCACGGTGTAAGGATG	[6FAM]CACTCCGTTGGTAAAAGAACTTGCACA[BHQ1]

Table S2. Primer and probe sequences for the respiratory multiplex Real-Time multiplex PCR. BHQ: Black Hole Quencher. Influenza A (generic and swine flu) from Carr et al[18]. All other sequences from Gunson & Carmen[17].

Virus target	Forward Primer Sequence 5′ - 3′	Reverse Primer Sequence 5′ - 3′	[Dye]ProbeSequence 5′ - 3′
Generic Influenza A	AAGACAAGACCAATYCTGTCACCTCT	TCTACGYTGCAGTCCYCGCT	FAM-TYACGCTCACCGTGCCCAGTG-BHQ
Influenza A H1N1 swine flu	TGTGCCACTTGTGAACAGATTG	CTGATTAGTGGATTGGTGGTAGTAGC	HEX-5′ TGATTCACAGCATCGGTCTCACAGACAG 3′-BHQ
Influenza B	ATGATCTTACAGTGGAGGATGAAGAA	CGAATTGGCTTTGRATGTCCTT	CY5-ATGGCCATCGGATCCTCAAYTCACTCT-BHQ
Human metapneumovirus B	GCYGTYAGCTTCAGTCAATTCAA (Common with A)	GTTATCCCTGCATTGTCTGAAAACT	VIC-CGCACAACATTTAGGAATCTTCT-MGBNFQ
Parainfluenza virus 1	GTGATTTAAACCCGGTAATTTCTCA	CCTTGTTCCTGCAGCTATTACAGA	FAM-ACCTATGACATCAACGAC-MGBNFQ
Parainfluenza virus 2	ATGAAAACCATTTACCTAAGTGATGGA	CCTCCYGGTATRGCAGTGACTGAAC	VIC-TCAATCGCAAAAGC-MGBNFQ
Parainfluenza virus 3	CCAGGGATATAYTAYAAAGGCAAAA	CCGGGRCACCCAGTTGTG	FAM-TGGRTGTTCAAGACCTCCATAYCCGAGAAA-BHQ
Parainfluenza virus 4	CAGAYAACATCAATCGCCTTACAAA	TGTACCTATGACTGCCCCAAARA	CY5-CCMATCACAAGCTCAGAAATYCAAAGTCGT-BHQ3A
Human coronavirus 229E	CAGTCAAATGGGCTGATGCA	AAAGGGCTATAAAGAGAATAAGGTATTCT	FAM-CCCTGACGACCACGTTGTGGTTCA-BHQ

Human coronavirus OC43	CGATGAGGCTATTCCGACTAGGT	CCTTCCTGAGCCTTCAATATAGTAACC	CY5-TCCGCCTGGCACGGTACTCCCT- BHQ
Human coronavirus NL63	ACGTACTTCTATTATGAAGCATGATATTAA	AGCAGATCTAATGTTATACTTAAAACTACG	VIC-ATTGCCAAGGCTCCTAAACGTACAGGTGTT-TAMRA
Respiratory syncitial virus A	AGATCAACTTCTGTCATCCAGCAA	TTCTGCACATCATAATTAGGAG	FAM-CACCATCCAACGGAGCACAGGAGAT-BHQ
Respiratory syncitial virus B	AAGATGCAAATCATAAATTCACAGGA	TGATATCCAGCATCTTTAAGTA	FAM-TTTCCCTTCCTAACCTGGACATA-BHQ
Rhinovirus	TGGACAGGGTGTGAAGAGC	CAAAGTAGTCGGTCCCATCC	VIC-TCCTCCGGCCCCTGAATG-TAMRA
Adenovirus	GCCACGGTGGGGTTTCTAAACTT	GCCCCAGTGGTCTTACATGCACATC	CY5-TGCACCAGACCCGGGCTCAGGTACTCCGA-BHQ

Table S3. Virological data from all 94 patients who had a nasopharyngeal swab taken. Data shown are cycle threshold (Ct) levels. No signal or values higher than 36 were considered negative (not shown in table). AdV: Adenovirus. EV: Enterovirus. FluA: Influenza A. FluB: Influenza B. HMV: Human metapneumovirus. PF!-3: Parainfluenzavirus 1, 2 and 3. RhV: Human rhinovirus. RSV: Respiratory syncytial virus.

Subjects	AdV	EV	FluA	FluB	HMV	PF1-3	RhV	RSV
**1**	-	-	-	-	-	-	-	-
**2**	33,1		-	-	-	-	-	-
**3**	-	-	-	34,9	25,4	-	-	-
**4**	31,1		-	-	-	-	-	-
**5**	-	31,8	-	-	-	-	-	-
**6**	-	26,2	-	-	-	-	-	-
**7**	-		-	-	-	-	-	-
**8**	-	-	-	-	-	-	-	32,9
**9**	-	-	-	-	-	-	21,1	33,0
**10**	14,5	-	-	-	-	-	-	-
**11**	-	-	-	-	-	-	31,8	-
**12**	-	-	-	-	-	-	33,4	-
**13**	-	-	-	-	31,2	-	29,0	-
**14**	-	-	28,5	-	-	-	-	-
**15**	-	-	-	-	35,7	-	-	-
**16**	-	-	-	-	-	-	-	-
**17**	32,8	-	-	-	35,4	-	-	-
**18**	-	-	-	-	-	-	-	-
**19**	-	-	-	-	-	-	30,2	-
**20**	-	-	-	-	-	-	-	-
**21**	35,3	-	-	-	-	-	-	-
**22**	-	-	33,9	-	-	-	-	-
**23**	-	-	-	-	34,3	-	-	-
**24**	-	-	-	-	-	-	-	-
**25**	-	-	-	-	33,8	-	25,4	-
**26**	-	-	26,1	-	-	-	-	-
**27**	-	-	-	-	-	-	-	-
**28**	-	-	-	-	-	-	-	-
**29**	-	-	-	-	-	36,0	-	30,6
**30**	-	-	-	-	-	-	-	-
**31**	-	-	-	-	-	-	34,6	-
**32**	-	-	-	-	-	-	-	-
**33**	-	27,3	21,8	-	-	-	-	-
**34**	-	-	-	-	-	-	-	-
**35**	-	-	27,5	-	34,6	-	-	-
**36**	-	-	-	-	-	-	-	-
**37**	-	35,6	-	-	-	32,3	-	23,4
**38**	-	27,3	-	-	-	-	26,3	34,9
**39**	-	-	-	-	-	-	-	-
**40**	-	-	-	-	-	-	35,8	-
**41**	-	-	-	-	-	-	-	-
**42**	-	-	-	32,1	-	29,9	-	-
**43**	-	-	-	-	-	-	-	-
**44**	-	-	-	-	-	-	-	31,1
**45**	-	-	-	-	-	-	-	-
**46**	-	-	-	-	-	-	30,0	-
**47**	-	-	-	-	-	-	-	-
**48**	-	-	-	29,3	-	-	-	-
**49**	-	-	-	-	-	-	-	-
**50**	-	-	-	-	35,2	-	-	-
**51**	-	-	-	-	-	-	-	-
**52**	-	-	-	-	-	-	25,5	-
**53**	-	-	-	-	-	-	-	-
**54**	-	28,2	24,6	-	-	32,5	31,6	-
**55**	34,9	35,9	-	-	-	-	30,3	-
**56**	-	-	-	24,8	-	-	-	-
**57**		-	-	34,7	-	-	-	-
**58**	24,1	-	-	-	-	-	-	-
**59**	-	-		-	-	-	-	-
**60**	-	-	-	-	-	-	-	-
**61**	-	-	-	35,0	-	-	-	-
**62**	-	-	-	-	-	-	-	-
**63**	-	-	-	-	-	26,1	-	-
**64**	-		-	-	-	-	-	-
**65**	-	-	-	-	-	-	24,5	-
**66**	-	34,4	-	-	-	-	31,2	-
**67**	-	-	-	-	-	-	-	-
**68**	-	-	-	29,4	-	-	-	-
**69**	-	-	-	-	-	29,4	-	-
**70**	-	-	-	-	-	-	-	-
**71**	-	-	-	-	31,7	-	-	-
**72**	-	-	-	-	-	-	-	-
**73**	-	-	-	-	-	-	-	34,9
**74**	-	24,8	-	-	-	-	-	33,8
**75**	-	-	-	-	-	-	-	23,1
**76**	-	29,6	-	-	-	-	29,7	-
**77**	-	-	-	-	-	-	-	30,9
**78**	-	-	22,3	-	-	-	-	34,4
**79**	-	-	-	-	-	-	25,7	25,5
**80**	-	-	-	-	-	-	-	24,1
**81**	-	-	-	-		-	-	-
**82**	-	-	-	-	-	-	-	30,0
**83**	-	22,3	27,5	-	-	-	-	-
**84**	-	-	-	-	-	-	-	-
**85**	-	-	31,2	-	-	35,9	-	-
**86**	-	-	-	-	-	-	-	32,9
**87**	23,9	-	-	-	-	-	-	-
**88**	-	-	-	-	-	35,3	-	-
**89**	-	-	-	-	-	-	-	27,1
**90**		-	29,8	-	-	-	-	31,5
**91**	-	-	-	-	-	33,8	-	-
**92**	22,9	-	-	-	-	-	-	-
**93**	-	-	-	35,9	-	-	-	-
**94**	-	-	-	-	-	-	-	-
